# Therefore, When I Grow Up I Want to Become an Aspirin Tablet

**DOI:** 10.3389/fpubh.2018.00169

**Published:** 2018-06-05

**Authors:** Ian A. Myles

**Affiliations:** Bacterial Pathogenesis Unit, Laboratory of Clinical Infectious Diseases, National Institute of Allergy and Infectious Diseases, United Stated Public Health Service Commissioned Corps, Bethesda, MD, United States

**Keywords:** Medical Education, Burnout, Professional, Empathy, purpose, Physicians

Interviewer: *Why do you want to be a doctor?*

Applicant: *To help people*.

This interview exchange is so cliché, it has become medical school lore. It has become such an expected question, followed by such a practiced answer, that schools and guidance councilors have given up on asking it all together—and medicine may be suffering for it.

There is a crisis among medical providers. A crisis that, even when discussed, seems relegated to articles far below the headlines. Far too many physicians hate going to work.

In the last scientific evaluation of practicing physicians, the field with the least dissatisfaction was infectious disease at 6.3% (1). Therefore, the specialty with the best disposition toward the field of medicine can proudly state that “only 1 out of every 16 of us wishes they had done something else with their life.” Overall dissatisfaction was nearly 1 in 5 (1). Nearly half of medical students experience burnout, meanwhile burnout among residents ranges from 18 to 82% (2). These are ominous findings for the preamble to a demanding profession. Accordingly, the reported physician burn out rate is upwards of 60% (3–5). Data on the rates of suicide among physicians is equally in need of updating but reported as over twice the rate of the general working population (6). Furthermore, the rate of suicide is higher among physician trainees, and was second only to cancer as a leading cause of death (6). Of perhaps greater concern, physician empathy for the people we are called to help is also far below ideal and worsened by distress (7, 8).

Being a physician is a distinctively difficult job but is not alone in its burden of stress and responsibility. Our US nursing counterparts also have suicide rates above national averages. Yet entire websites have emerged as soundboards for gripes about being a physician as well as grievances against the patients we profess to have been called to help. So why might physicians have such issues with satisfaction and empathy? No can provide a data-driven answer to such a question. Thus, this essay does not intend to make a 1-to-1 connection between failure to examine why one wants to become a physician and eventual job satisfaction. Rather, it hopes to open discussion on the cause of our high job dissatisfaction and to query the role of pre-medical school preparation.

Job dissatisfaction was not discussed at any point in my medical training. However, the question came to the forefront reviewing applications for a national health scholar program where those accepted receive paid medical school tuition in exchange for service to federal health programs. A clear pattern formed in the narratives essays included in the application: the first part was “how I learned diseases cause suffering,” followed by “how I learned doctors reduce disease.” Part one was a story about a loved one that fell ill and the subsequent pain and sorrow the illnesses caused. In the second part the story narrated tales of a mentor physician, an M.D. parent, or the provider treating the aforementioned loved one. In no way do I mean to make light of these stories or their subsequent realizations. Nor do I wish to imply that the life lessons taken from them are not vital parts of pre-medical school education. However, their premises do not lead to the conclusion that medical school attendance is the appropriate next step. Translated into Socratic logic this thinking reads:

Disease increases suffering
Doctors can reduce disease
Therefore, doctors can reduce suffering

An inspirational epiphany for those hoping to better the world, but not one that assures becoming an M.D. is the best path. The logical fallacy that is derived from most of the applications would be:

Disease increases suffering
Doctors can reduce disease
Therefore, when I grow up I want to become a doctor

Nurses are essential to reduce suffering, as are therapists, pharmacists, and environmental health officers. Even medical administrators and medical billers are essential components to the coordinated effort to combating disease and thereby reducing suffering. Indeed, many in these other health professions may have also been motivated into health care by witnessing disease.

Shut the ER down and waiting rooms will clog and quality of care will decrease, shut down the OR and build up may transform urgencies into emergencies, but in either case the hospital will not crumble. But shut down the modern hospital's IT department and the entire building bewilders into pre-enlightenment medicine. Yet, no one applies for a hospital IT job with a page of proses about combating suffering.

We have forgotten to ask why applicants want to become a *doctor*, instead asking only why they want to provide medical assistance. If they envision providing care hands on, the hectic pace of hospital medicine may not be as fulfilling as the one-on-one of physical therapy. If their heart is drawn to widespread impact, perhaps the field of public health policy is their true calling. Primary care allows for impacting the very foundation of patient health and affords protracted follow-up to see a child blossom and a family grow. However, pay is often lower than other specialties, especially when compared to the ever-increasing average student loan debt. Private practice affords freedom of decision-making and professional autonomy but carries with it all the troubles of being both a medical provider and a small business owner. Surgery can provide immediate gratification but also immediate disappointment if patients do not survive in spite of intervention. Research and public health allow for large-scale improvements in the standard of care, but must suffer at a bureaucratic pace, publish-or-perish pressures, with funding always below optimal. If the applicant knows they yearn for the excitement of bringing patients back from certain death in the trauma bay, then stating such outright allows for honest assessment of the pros and cons of the job. This introspection could foster re-focusing on one's purpose when times become challenging. Thus the question needs to be not only a thorough introspection on why one would want to be an M.D., but an inquiry into what we generally envision for our scope of practice. With interview season once more upon us, lets ask this vital question no matter how cliché. Not entirely to challenge the applicant as to their true intent, but to cement the reasoning in their mind as to levee against the floods of challenges that will befall them. Failure to ask why the path to an M.D. is best for the applicant is a disservice to future physicians and the specialty as a whole.

By extension this question should be asked at every level of training; articulating an honest discussion about what draws one to a particular specialty. One of the key distinguishers of burnout from exhaustion and compassion fatigue (depersonalization) is that burnout typically includes a failure to recognize the purpose in one's work (loss of professional efficacy) (9). Many are willing to work themselves into fatigue so long as they see their labors as serving a purpose, however the feeling of “what's the point of all this?” seems to indicate the onset of burnout. Thus, perhaps fostering clear articulation and continued focus on what the guiding purpose for a trainee's career choice might inoculate against decisions that increase burnout. As hypothetical example, assume a future physician knows early that she wants to specialize in emergency medicine for the fast-pace and chance to treat high-acuity patients. Years later if offered a position with better pay and better hours at a small, slow-paced, community ER with no trauma designation, perhaps she would either reflect on whether her reason for enjoying emergency medicine has changed or would attempt to find ways to keep trauma response as part of her life.

However, some may put forth an alternative hypothesis: perhaps asking this question has little value because twenty-some years of life are incapable of truly informing one's decision to become a physician. Perhaps no generation of physicians ever truly knew what they were getting into when setting foot on their med school campus for the first time. To believe this, we would have to either assume that two decades fail to prepare physicians but adequately prepare for careers in other health professions; alternatively, we would have to assume nearly the entire health field could not articulate the reasons for their chosen career path. Either explanation represents a failure of the medical education community to best prepare applicants for the consequences of their enrollment.

Rationalizing away this failure by suggesting that inexperience precludes introspection displays a lack of concern for a process that commits young adults to a decade of training and significant financial debt, followed by a highly stressful career. Enabling such commitments despite believing the students have little insight into their decision seem unlikely to benefit the student or the profession. We should not be comfortable asserting that becoming a physician is a decision that we as educators simply cannot prepare students for. Thus, I feel that new approaches should be formulated as to how to improve our pre-med preparation. The reasons for dissatisfaction among physicians and students are clear: debt, regulations, sleep deprivation, loss, exams, certifications, and bureaucracy can readily leech one's enjoyment of medicine (8, 10, 11). We know what goes wrong. What medicine needs is a mechanistic study on satisfaction; uncovering what the happy practitioners believe the cause of their satisfaction to be and how their field meshes with their personality. In so doing, we could reverse-engineer a Myers-Briggs type evaluation that could provide applicants a better understanding of how the field will treat them. Thankfully, many new approaches to treat and prevent burnout are being deployed, including mindfulness training, and stigma-removing open dialogue about burnout and its causes (11, 12).

As best as possible, formulating precisely why and how an applicant plans to practice medicine would at least partially inoculate against losing their grasp of their noble goals and from distractions detouring them from their true motivations. Thus, admission committees as well as college pre-med advisors should encourage thoughtful introspection and refuse to accept practiced and banal responses. This change would help medical applicants understand the trials of medical practice and perhaps thereby maintain empathy for the populations they seek to serve. Addressing low job satisfaction among physicians will certainly take more than a med-school application questionnaire. Yet, the need to confront it is clear and perhaps a better understanding of our driving motivations may allow for a better focus on protecting those noble intentions from the realities of the profession.

## Author contributions

The author confirms being the sole contributor of this work and approved it for publication.

### Conflict of interest statement

The author declares that the research was conducted in the absence of any commercial or financial relationships that could be construed as a potential conflict of interest.
